# Predictors of Adherence to Type 2 Diabetes Medication 

**Published:** 2016-05-20

**Authors:** Azar Pirdehghan, Nafise Poortalebi

**Affiliations:** ^a^ Department of Community and Preventive Medicine, School Of Medicine, Hamadan University of Medical Sciences, Hamadan, Iran; ^b^ Department of Community and Preventive Medicine, School Of Medicine, University of Shahid Sadoughi, Yazd, Iran

**Keywords:** Diabetes Mellitus, Adherence, Diabetes, Medication Adherence, Health Beliefs

## Abstract

**Background:** Despite the effectiveness of drug therapy in diabetes management high rates of poor adherence persist. The purpose of this study was to determine the factors influencing the medication adherence and dietary regiment in type2 diabetic patients.

**Methods:** This cross sectional study was conducted on 300 type2 diabetic patients referred to General Internal Medicine Clinic, Yazd Shohdaye Kargar Hospital, Yazd City, central Iran between September and December 2013. Each consented participant was interviewed by a trained study member using a questionnaire in three sections: Socio-demographic questions, self-reported Morisky medication adherence scale and Disease and medication beliefs Patient’s questionnaire. Multivariable logistic regression model was developed to identify independent predictors of poor adherence. P<0.05 was considered statistically significant.

**Results:** Patients had diabetes for an average of 8.87 (SD: 6.0) yr with a mean age of 58.22 (SD: 10.27) yr. Totally, 101(33.7%) of the patients reported poor adherence with their diabetes medication. In multivariate analyses, good familial support (OR=0.11; 0.03, 0.37), and tendency to consume sweets (OR=1.21; 1.05, 1.39), belief about medication (OR=0.02; 0.018, 0.07) and tendency to consume vegetables (OR=0.75; 0.65, 0.88) were considered as predictive factors for poor adherence.

**Conclusions:** Familial support, belief about medication, tendency to consume sweets and vegetables are logical goals for educational interventions to modify diabetes self-management.

## Introduction

Diabetes is one of the most increasing common public health issues globally. This disease known to be the fifth cause of mortality in the western societies^1^and fourth reason of referring to the physicians, has been allocated 15% of health care costs in the USA^2^.

The incidence rate of diabetes type 2 is increasing in the world which can be due to life style change resulting in obesity and reduction of physical activity. The incidence of diabetes in Iran is accounted for 14% in the population over 30^3^. Training diabetic patients along with adherence allows the patients to take more responsibility to care themselves^4^. Adherence is the amount of acceptance and adaptability with caregivers’ advice^5^. According to WHO, adherence includes the change in individual`s behavior, drug administration, regiment follow-up, life style change and application of caregivers’ advice^6^. For 36%-93% of cases, medication adherence by diabetic type 2 patients is not satisfactory^7^. There have been some discussions about the effects of weak adherence such as weak control of blood sugar and preventable hospitalization in chronic diseases such as diabetes and blood pressure^8-10^.

Different studies have mentioned several factors low of medication adherence such as the number of administered medications, complexity of treatment regiment, comorbidity, weak family support and inaccessibility to medication or lack of insurance coverage for the required drugs^6,11-13^.

Regarding the importance of adherence in management of diabetic patients, we aimed to determine the factors influencing the medication adherence and dietary regiment in type 2 diabetic patients so that there can be suggested some strategies to plan interventions.

## Methods

This cross sectional was study in non-randomized sampling method conducted on 300 type2 diabetic patients referred for control and complication follow up. After Institutional Review Board approval, study participants were recruited from General Internal Medicine Clinic, Yazd Shohdaye Kargar Hospital, Yazd City between September and December 2013. Trained staff identified patients using a list of adults with diabetes coming in for visits each day and approached these potential participants in the waiting room. All 30- yr- old or older patients reporting a history of type 2 diabetes for at least 12 months prescribed diabetes medication were enrolled. Exclusion criteria included patient who refused insulin therapy recommendation and stayed on drugs for different reasons.

Each consented participant was interviewed by a trained study member using a questionnaire in three sections: Socio-demographic questions, self-reported Morisky medication adherence scale and Disease and medication beliefs Patient’s questionnaire.

Socio-demographic factors, diabetes history, and co morbidities (medical and psychiatric) were self-reported. The primary outcome measure of adherence to diabetes medicines was determined using a modified version of the six items, self-reported Morisky medication adherence scale^14^. Each item is in a yes/no format with possible score of four to six, equating good adherence and zero to three typically considered as poor adherence. The Morisky scale has been used across many chronic diseases, including diabetes, as a self-reported measure of adherence to medications and has demonstrated good reliability and predictive validity^15-17^. In present study, it had proper reliability (Cronbach alpha: necessity=0.70).

Disease and medication beliefs Patient's disease beliefs were measured with 19 item assessing beliefs about the chronicity, cause, consequences and controllability of their diabetes using the Brief-Illness Perception Questionnaire (test–retest reliability across domains (0.42 to 0.72) as a framework^18^.

Medication beliefs were assessed using the five items of greatest relevance to diabetes medication adapted from the Beliefs about Medicines Questionnaire (Cronbach alpha: necessity=0.74, concerns=0.80)^19^.

Additional questions assessed familial support and access to medicine in tree scale (poor, moderate and good) and tendency to eat fruits vegetables, meat and sweetmeat in score rang 1 to 10.

Categorical variables are reported as percentages and continuous variables as means. Socio-demographics, medical history, beliefs and knowledge rates were calculated using descriptive statistics. The final multivariable logistic regression model was developed to identify independent predictors of poor adherence based on the variables associated with adherence in the univariate analysis using a Forward conditional model. All statistical analyses were performed using SPSS.19 statistical software (Chicago, IL, USA). *P*<0.05 was considered statistically significant.

## Results

The 300 study subjects were all clinic attending patients with type 2 diabetes of age between 30 and 85 yr. More than 50% of participants had longstanding diabetes (7 yr and more) and more participants were on medications included in glibenclamide or metformin.

Respondents reported high levels of co morbid conditions (hypertension 189 (63%) and hyperlipidemia 171 (57%)) commonly associated with diabetes (Table1). Totally, 101 (33.7%) of patients reported poor adherence with their diabetes medication.

Among demographic and clinical variables analyzed, being single or divorced, drug medication versus insulin therapy, younger age and lower duration of disease had a significant relationship with poor adherence in diabetic patients (*P*<0.05) (Table 1 and 2).

**Table 1:** Socio-demographic and clinical characteristics of study patients (n=300)

**Categorical variables**	**Fine adherence**	**Poor adherence**	***P*** ** value**
	**Number (%)**	**Number (%)**	
Gender			0.269
Female	78 (70.3)	33 (29.7)	
Male	121 (64.0)	68 (36.0)	
Marital status			0.007
Married	180 (69.2)	80 (30.8)	
Single or divorce	19 (47.5)	21 (52.5)	
University education			0.490
Yes	31 (67.4)	15 (32.6)	
No	168 (66.1)	86 (33.9)	
Family history of diabetes			0.043
Yes	45 (77.6)	13 (22.4)	
No	154 (63.6)	88 (36.4)	
Medication type			0.003
Insulin	46 (78.0)	13 (22.0)	
Drug	153 (64.6)	84 (35.4)	
Hypertension			0.054
Yes	133 (70.4)	56 (29.6)	
No	66 (59.5)	45 (40.5)	
Hyperlipidemia			0.378
Yes	117 (68.4)	54 (31.6)	
No	82 (63.6)	47 (36.4)	
**Continuous variables**	**Mean (SD)**	**Mean (SD)**	***P*** ** value**
Age (yr)	59.42 (10.01)	55.84 (10.39)	0.004
Diabetes duration (yr)	9.78 (6.24)	7.07 (5.05)	0.001

**Table 2:** The comparison of Morisky score between different situation of some familial environment adherence related factors

**Variables**	**Number (%)**	**Morisky Mean (SD)**	***P*** ** value**
Familial support			0.001
Poor	28 (9.3)	2.57 (1.57)	
Moderate	74 (24.6)	3.68 (1.83)	
Fine	198 (66.0)	4.17 (1.56)	
Effects of family disease related advises	0.001
Poor	29 (9.6)	2.30 (1.89)	
Moderate	72 (24.0)	3.86 (1.75)	
Fine	197 (65.6)	4.14 (1.53)	
Accessibility to medications		0.001
Poor	10 (3.3)	2.40 (1.64)	
Moderate	62 (20.6)	3.22 (1.91)	
Fine	225 (75.0)	4.15 (1.55)	

Across some social factors, poor familial support, family disease related advises and insufficient accessibility to medications were significant related agents with poor adherence (*P*<0.05) (Table 2).

Based on the results of simple logistic regression, significantly related risk variables for "poor adherence", were candidate as to enter in the multivariate analysis model. Among them poor familial support and tendency to consume sweets were considered as predicting and belief about medication and tendency to consume vegetables as protective factors (Table 3).

**Table 3:** Logistic regression analysis of the relationship between poor adherence and risk variables based on forward conditional procedures

**Variables**	**Odd ratio (95% CI)**	***P*** ** value**
Familial support		
Poor	1.00	
Moderate	0.21 (0.06, 0.70)	0.011
Good	0.11 (0.03, 0.37)	0.001
Tendency to consume sweets	1.21 (1.05, 1.39)	0.009
Tendency to consume vegetables	0.75 (0.65, 0.88)	0.001
Belief about medication	0.02 (0.01, 0.07)	0.001

## Discussion

66.3% of patients had acceptable medication adherence. These were varied in other studies. For example, in Malaysia, aiming to investigate the medication adherence and the factors related to non-adherence to treatment on 557 type 2 diabetic patients, 53% of patients had unfavorable medication adherence^20^. In another study in France, aiming to evaluate self-reporting medication adherence and factors related to weak medication adherence on type 2 diabetic patients, 12% of patients had weak medication adherence, 49% medium and 39% had good adherence^21^.

The difference of results in studies can be related to the design of studies, demographic differences of the subjects, different methods in investigating medication adherence and different situation of training patients. In our study, the frequency of patients under study along with unfavorable medication adherence was underestimated due to the following reasons: First, the determination of medication adherence was based on asking the patients, therefore, some patients might answer the questions in favor of the interviewer making the medication adherence unreal. Second, the probability of volunteer bias must have been considered in our study. Surely, those who regularly refer to control blood sugar and visit the physician in health centers have better medication adherence at home and apply the physicians` advice. Some others who are not included in our study due to non-referral to health centers had less medication adherence.

In this study, there was a significant difference between the time of diabetes diagnosis and age with medication adherence in that age group over 75 yr showed higher adherence than other groups. In a study in Tehran, on type 2 diabetic patients for their medication adherence in the last month and their awareness of diabetes, symptoms and attitude on the necessity of medication, subjects over 45 yr showed higher medication adherence^22^.

Some studies have regarded the medication adherence in patients over 75 yr due to the memory disorder and movement, visual and depression disorder lower than the younger ones inconsistent with our study^23^. The significant relationship between age and medication adherence in our study is due to the fact that the elderly see themselves more vulnerable to death, dependency on the family and the symptoms of disability.

In this study, there was a significant relationship between marital status and medication adherence so that the married subjects showed greater adherence than two other groups, as consistent with another study^24^.

In present study, there was a significant relationship between medication adherence with marital status, family support and access to drug in that medication adherence in subjects with good family support and access to drug was remarkably greater. In another study, factors related to weak medication adherence in patients over 45 yr were lack of family support and weak access to drug^21^. In England, non-supportive behaviors of family were related to weak diabetic medication adherence and reduced lowering blood sugar^25^.

Our results showed that in subjects believing the disease and stronger treatment, the medication adherence was greater. False belief was the barrier in control and behavior management and consequently the weak medication adherence of patients^26^.

In this study, there was a significant relationship between medication adherence and dietary regiment with patient’s efficaciosity to family members’ advice to suitable regiment in that they had more adherence. In Botswana, lack of admission of advice related food regiment and sports led to non-admission of food regiment and disease exacerbation, as consistent with the present study^27^.

Our results should be viewed with consideration of limitations. While our study population was only the patients who referred to general internal medicine clinic in order to follow the complications of their disease, the generalizability of our observations to other settings is unknown, and should be explored in future work. And maybe in our research more number of sample sizes could detect weaker associations.

## Conclusions

More than 30% of diabetic patients had unacceptable adherence. In multivariate analyses, poor familial support, and tendency to consume sweets were considered as predicting and belief about medication and tendency to consume vegetables as protective factors. Maybe the mention above could be considered as logical goals for educational interventions to modify diabetes self-management in future programs.

## Acknowledgments

We gratefully acknowledge all health professionals staff in Yazd Shohdaye Kargar Hospital General Internal Medicine Clinic that helped for data collection.

## Conflict of interest statement

The authors declare that have no conflict of interests.

## Highlights

- More than 30% of diabetic patients had unacceptable adherence.
- Poor familial support is a predicting factor for poor adherence in diabetic patients.
- Medication belief is a protective factor for poor adherence in diabetic patients.
